# Expression of *Cyr61*, *CTGF*, and *WISP-1* Correlates with Clinical Features of Lung Cancer

**DOI:** 10.1371/journal.pone.0000534

**Published:** 2007-06-20

**Authors:** Ping-Ping Chen, Wen-Jie Li, Yan Wang, Song Zhao, De-Yun Li, Li-Yun Feng, Xiang-Lin Shi, H. Phillip Koeffler, Xiang-Jun Tong, Dong Xie

**Affiliations:** 1 Laboratory of Molecular Oncology, Institute for Nutritional Sciences, Shanghai Institutes of Biological Sciences, Chinese Academy of Sciences, Graduate School of the Chinese Academy of Sciences, Shanghai, China; 2 College of Public Health, Zhengzhou University, Zhengzhou, China; 3 Department of Surgery, the First Affiliated Hospital of Zhengzhou University, Zhengzhou, China; 4 Institute for Medicine of Chronic Disease, Disease Control and Prevention of Sichuan, Sichuan, China; 5 Department of Hematology and Oncology, Cedars-Sinai Medical Center, University of California Los Angeles (UCLA) School of Medicine, Los Angeles, California, United States of America; 6 College of Life Sciences, Peking University, Beijing, China; University of Minnesota, United States of America

## Abstract

**Background:**

CCN family, comprising six members (Cyr61, CTGF, Nov, WISP-1, WISP-2, WISP-3), is involved in the stimulation of cell proliferation, migration, adhesion, angiogenesis, and tumorigenesis. Several studies have shown that expression of Cyr61, CTGF, and WISP-1 affects the tumorigenic potential of lung cancer cells in vitro. However, the correlation of expression of CCN family proteins and clinical features of lung cancer remains unknown.

**Methodology and Principal Findings:**

In the present work, we quantified the mRNA levels of *Cyr61*, *CTGF*, and *WISP-1* in samples from 60 primary lung cancers and their matched normal lung tissues by quantitative real-time PCR assay. Downregulation of the *Cyr61* and *CTGF* genes and upregulation of the *WISP-1* gene were found in primary lung cancers compared to the paired normal lung tissues. Immunohistochemistry analysis also disclosed a similar expression pattern of Cyr61, CTGF, and WISP-1 protein in paired lung cancer tissues. Statistical analysis revealed significant associations between expression of either *Cyr61* or *CTGF* with tumor stage, tumor histology, metastasis, smoking, and family history at diagnosis. A significant correlation also existed between WISP-1 expression with tumor histology, and patient age. Moreover, expression levels of *Cyr61* and *CTGF* correlated with survival of the lung-cancer patients.

**Conclusions:**

Our results suggest that *Cyr61*, *CTGF*, and *WISP-1* might be implicated in the development and progression of primary lung cancers, and their levels might serve as valuable prognostic markers, as well as potential targets for therapeutic intervention.

## Introduction

Lung cancer is the most common cause of cancer death in the world [1]. The majority of lung cancers are non-small-cell lung carcinoma (NSCLC), which is subdivided into adenocarcinoma (AC), squamous-cell carcinoma (SC), and large-cell carcinoma [2]. The high mortality associated with NSCLC is in part due to metastasis before surgical removal of the primary tumor. Tumor metastasis involves detachment of tumor cells from the primary tumor mass, microinvasion of tumor cells into stromal tissue, intravasation of tumor cells into blood vessels, and extravasation and growth of tumor cells in secondary sites [3], [4]. To become metastatic, tumor cells must increase the expression of metastasis-promoting genes and/or decrease the expression of metastasis-suppressing genes. Utilizing human lung cancer cell lines, cysteine-rich protein 61 (Cyr61) and connective tissue growth factor (CTGF) have been demonstrated to inhibit metastasis and invasion of cancer cells, and therefore they have been considered as potential suppressors of metastasis [5], [6].

Both CTGF and Cyr61 belong to the CCN family, named for its three first described members - **C**yr61 (CCN1), **C**TGF (CCN2), and **N**ov (nephroblastoma overexpressed, CCN3) [7]. CCN family has six members : Cyr61, CTGF, Nov, WISP-1 (Wnt-1-induced secreted protein 1) (CCN4), WISP-2 (CCN5) and WISP-3 (CCN6) [8]–[11]. These proteins are modular in structure, consisting of an N-terminal signal sequence followed by domains with sequence similarity to insulin-like growth factor-binding protein, von Willebrand factor C, thrombospondin type 1, and a cysteine knot at the C terminus with an exception of WISP-2, which lacks the C terminus region [12]. All CCN molecules are secreted, extracellular matrix-associated proteins and involved in internal and external cellular signaling to regulate cell adhesion, migration, mitogenesis, differentiation, and survival [13]. They also regulate angiogenesis [13]–[15]. Previous studies suggested that Cyr61 induces cell proliferation, cell adhesion and angiogenesis through activation of integrin (αVβ3) in endothelial cells [16]. CTGF plays a key role downstream of TGF-β and SMAD signaling and stimulates production of fibronectin and collagen, which is important for wound healing [17], [18]. WISP-1 is strongly expressed in the fibrovascular stroma of breast tumors developing in Wnt-1 transgenic mice [10]. Forced overexpression of WISP-1 in normal rat kidney fibroblasts (NRK-49F) was sufficient to induce their transformation [11]. Moreover, increasing evidence has suggested that CCN proteins are involved in tumorigenesis, and variation of expression of these molecules has been observed in several types of cancers [8], [9].

We have demonstrated that Cyr61 acted as a tumor suppressor in the growth of NSCLC cells. Cyr61 suppressed the growth of NSCLC cells by triggering a signal transduction pathway through upregulation of p53 [6], [19]. CTGF and WISP-1 also affected the tumorigenicity of lung cancer cells [5], [20]–[22]. However, correlations between the three molecules with clinical features of lung cancer are unexplored. Our previous work suggested that expression of CCN family proteins has prognostic value for glioma progression and overall patient survival [23]. In the present studies, we performed real-time quantitative RT-PCR and immunohistochemistry to measure the mRNA and protein levels of three CCN genes in primary NSCLC samples and their matched normal lung tissues. Furthermore, we determined whether levels of these CCN genes were correlated with clinical features of NSCLC samples by several statistical analysis models.

## Materials and Methods

### Patients and samples

This study analyzed the primary cancer and matched normal tissues from 60 NSCLC patients treated at the First Affiliated Hospital of Zhengzhou University (Henan, China) from 2002 to 2005 after their written informed consent. Cancer samples were resected surgically without any neo-adjuvant therapy and corresponding non-cancerous tissues, which were at least 3–4 cm away from cancer, were also obtained. Each specimen was divided into 2 parts: one was sectioned and examined histologically by traditional H&E staining for the presence of more than 80% tumor cells (cancer sample) or only normal cells without any inflammatory or tumor infiltrating areas (matched normal sample); the other was frozen in liquid nitrogen and stored at −80°C until analysis. Our work was approved by the Institutional Review Board of the Institute for Nutritional Sciences, Chinese Academy of Sciences.

### RNA extraction and cDNA synthesis

Total RNA was extracted from fresh-frozen NSCLC specimens and matched normal lung tissues by TRIzol reagent (Life Technologies, Inc.) according to the manufacturer's protocol. The quality of the RNA samples was determined by electrophoresis through agarose gels and staining with ethidium bromide, the 18S and 28S RNA bands were visualized under UV light. 2 µg of total RNA was processed directly to cDNA by reverse transcription with Superscript II (Life Technologies, Inc.) according to the manufacturer's protocol in a total volume of 50 µl.

### Real-time reverse transcription PCR (RT-PCR)

RT-PCR was characterized at the point during cycling when amplification of the PCR product was first detected, rather than the amount of PCR product accumulated after a fixed number of cycles. The parameter Ct was defined as the fractional cycle number at which the fluorescence generated by passing a fixed threshold above baseline. The level of target gene in unknown samples was quantified by measuring the Ct value: Level(target) = 2^Ct(target)^. The Ct value of β-*actin* was also measured as the endogenous RNA control: Level(β-*actin*) = 2^Ct(β*-actin*)^. The levels of target genes in each sample were normalized on the basis of its β*-actin* content through the formula: Normalized level (NL) = Level(target)/Level(β-*actin*) = 2^Ct(target)^/2^Ct(β-*actin*)^ = 2^Ct(target)-Ct(β-*actin*)^ = 2^ΔCt^. Furthermore, the relative levels (RL) of target genes in cancer samples versus matched normal tissues were calculated according to the formula: RL = NL(cancer)/NL(normal) = 2^ΔCt(cancer)^/2^ΔCt(normal)^ = 2^[ΔCt(cancer)−ΔCt(normal)]^ = 2^ΔΔCt^. Because both NL and RL are represented as 2^Ct^, we therefore used ΔCt and ΔΔCt as NL and RL, respectively, for clinical statistical analysis.

Primers for real-time PCR for *Cyr61* (5′-GAGTGGGTCTGTGACGAGGAT-3′ and 5′-GGTTGTATAGGATGCGAGGCT-3′), *CTGF* (5′-CGACTGGAAGACACGT TTGG-3′ and 5′-AGGCTTGGAGATTTTGGGAG-3′), *WISP-1* (5′-AGAGCCGC CTCTGCAACTT-3′ and 5′-GGAGAAGCCAAGCCCATCA-3′) and β-*actin* (5′-G ATCATTGCTCCTCCTGAGC-3′ and 5′-ACTCCTGCTTGCTGATCCAC-3′) were designed using software PRIMER3 and purchased from Shanghai Sangon Biological Engineering Technology & Services CO., Ltd. Amplification reactions were performed in a 20 µl volume of the LightCycler-DNA Master SYBR Green Ι mix (Roche Diagnostics Ltd., Applied Science, Penzberg, Germany) with 10 pmol of each primer, MgCl_2_ concentration optimized between 2 and 5 mM, 200 µΜ of each dNTP, 0.5 U Taq DNA polymerase, and 1×buffer. All of the reactions were performed in triplicate in an iCycler iQ system (Bio-Rad, Hercules, CA), and initial denaturation at 95°C for 3 min was followed by 40 cycles of a denaturation at 95°C for 30 s, an annealing step between 55°C and 58°C for 20 s, and an extension step at 72°C for 30 s. A final extension step at 72°C for 7 min was added. To confirm specificity of amplification, the PCR products from each primer pair were subjected to a melting curve analysis and subsequent inspection after agarose gel electrophoresis.

### Statistical analysis

T-test and ANOVA were adopted to study the expression of 60 pairs of NSCLC and normal lung tissues for each CCN gene and their association with single clinical factors (family history, metastasis, smoking, tumor stage, histology, tuberculosis, gender, tumor size and patient's age). Pearson's correlation analysis was used to estimate relative degree by quantity of the expression of the three genes. Pearson's correlation reflects the degree of linear relationship between two variables. It ranges from +1 to −1. The relative values (tumor/control) were relative to actin plotted. For each gene, Kaplan-Meier survival curves for patients with high gene expression *versus* low gene expression were plotted and log-rank test was used for comparing the equality of the two survival curves. Multivariable analyses with the Cox proportional hazards model were used to estimate the effects of the clinical characteristics and the expression of the three genes on survival. Results were considered significant at P<0.05 or highly significant at P<0.01. All statistical analyses were performed using the program SPSS for Windows (SPSS, Chicago, IL).

### Immunohistochemistry

For immunohistochemistry, cancerous and corresponding normal lung tissues were frozen in a cryostat chamber and 10 µm sections were collected on glass slides. The sections were fixed in ice-cold acetone for 30 min, washed in 0.01 M PBS for 3 × 5 min, blocked for 1 hr in 0.01 M PBS supplemented with 0.3% Triton X-100 and 5% normal serum, and then incubated with rabbit anti-human Cyr61 (polyclonal, 1∶500; Santa Cruz Biotechnology, Santa Cruz, CA, USA), goat anti-human CTGF (polyclonal, 1∶500; Santa Cruz Biotechnology, Santa Cruz, CA, USA), or goat anti-human WISP-1 (polyclonal, 1∶500; Santa Cruz Biotechnology, Santa Cruz, CA, USA), respectively, at 4°C overnight. After brief washes in 0.01 M PBS, sections were incubated for 2 hr in 0.01 M PBS with horseradish peroxidase-conjugated goat anti-rabbit IgG or rabbit anti-goat IgG (1∶1000; Chemicon, Temecula, CA, USA), followed by visualization with 0.003% H_2_O_2_ and 0.03% DAB in 0.05 M Tris-HCl (pH 7.6). Negative controls consisted of substitution of the primary antibody with normal serum at the same dilution. Immunohistochemistry for each individual was performed at least 3 times and all sections were counterstained with hematoxylin. Scoring of immunohistochemistry staining was carried out independently by three pathologist blinded to the patient's clinical parameters. All stained sections were scored both in the tumor and adjacent non-tumor areas at least in 10 high-power field areas with a minimum of 300 preserved cells assessed in each area. The percentage of cells expressing target protein was estimated by dividing the number of positive cells by the number of total cells per high-power field area. Lung epithelial cells bearing obvious brown signal in the cytoplasm compared with negative control were defined as positive cells. Paired-samples t-test was used to evaluate the difference between protein levels of Cyr61, CTGF, or WISP-1 in cancer samples compared with matched normal lung tissues. Results were considered statistically highly significant at P<0.01. All statistical analyses were performed using the program SPSS for Windows (SPSS, Chicago, IL).

## Results

### Expression of *Cyr61*, *CTGF*, and *WISP-1* genes in NSCLC and matched normal lung tissues

**Figure 1:**
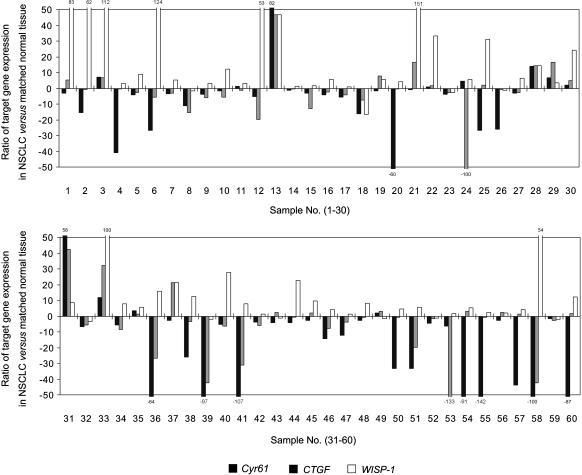
Expression patterns of *Cyr61*, *CTGF*, and *WISP-1* mRNAs in NSCLC and matched normal lung tissues. Relative mRNA expression levels of *Cyr61*, *CTGF*, and *WISP-1* are shown in 60 pairs of primary NSCLC tissues and matched normal lung samples. Expression is displayed as a ratio of expression of CCN genes in cancer *versus* matched normal tissues. Each bar is the log2 value of the ratio of CCN expression levels between lung tumors (T) and matched normal tissues (N) from the same patients. Less than 2-fold change: the ratio between tumor and normal is <2. Because Log2 2 = 1, bar value >1 represents >2-fold increase (T>N), whereas bar value <−1 represents >2-fold decrease (T<N).

To study the expression pattern of CCN genes in NSCLC, levels of *Cyr61*, *CTGF*, and *WISP-1* mRNA were quantified in 60 pairs of tumors and their matched normal lung tissues by real-time PCR. Expression level was shown as a ratio between *Cyr61*, *CTGF*, or *WISP-1* and the reference gene β-*actin* to correct for the variation in the amounts of RNA. Downregulation of *Cyr61* and *CTGF* mRNA occurred in 48 of 60 (80%) and 39 of 60 (65%) NSCLC samples compared with the paired normal lung tissues, respectively. In contrast, upregulation of levels of *WISP-1* mRNA was observed in 50 of 60 (83%) NSCLC samples in comparison with their normal tissues (Fig. 1). Univariate analysis showed that mRNA level of either *Cyr61*, *CTGF*, or *WISP-1* genes were significantly different between the cancer samples and paired normal ones (Table 1). Expression of *Cyr61* and *CTGF* in primary lung cancers was significantly lower than in matched normal lung tissues (P<0.001 and P = 0.016, respectively). In contrast, levels of *WISP-1* in cancers were significantly higher than those in the matched normal ones (P<0.001) (Table 1). Pearson's correlation analysis further showed that expression of *Cyr61* and *CTGF* was highly positively correlated (R = 0.604; P<0.001), whereas expression of *WISP-1* and *CTGF* showed a significant negative association (R = −0.299; P = 0.020). Expression of *WISP-1* and *Cyr61* was not significantly correlated (R = −0.182; P = 0.164) (Table 2). Because the three genes are all members of CCN family, it is possible that their expression might be controlled by some common regulators (for *Cyr61* and *CTGF*) or antagonized by each other (for *WISP-1* and *CTGF*).

**Table 1:** Expression of *Cyr61*, *CTGF*, and *WISP-1* mRNA in lung cancer and matched normal lung tissues.

Gene	N	Cancer	Matched-normal	P‡
*Cyr61*	60	2.74±4.16†	4.93±3.34	0.001
*CTGF*	60	1.40±3.53	2.41±3.37	0.016
*WISP-1*	60	−3.09±3.53	−5.82±3.67	0.001

† Means ± SD of expression of *Cyr61*, *CTGF*, and *WISP-1* mRNA in lung cancer or matched normal samples after normalized to β*-actin* in the same samples.

‡ P<0.05 and P<0.01 are set for significant and highly significant difference, respectively.

**Table 2:** Pearson’s correlation matrix of *Cyr61*, *CTGF*, and *WISP-1* mRNA in 60 pairs of samples.

Variables	Correlation (R)	P†
*Cyr61* and *CTGF*	0.604	0.001
*Cyr61* and *WISP-1*	−0.182	0.164
*CTGF* and *WISP-1*	−0.299	0.020

† P<0.05 and P<0.01 are set for significant and highly significant difference, respectively.

**Figure 2:**
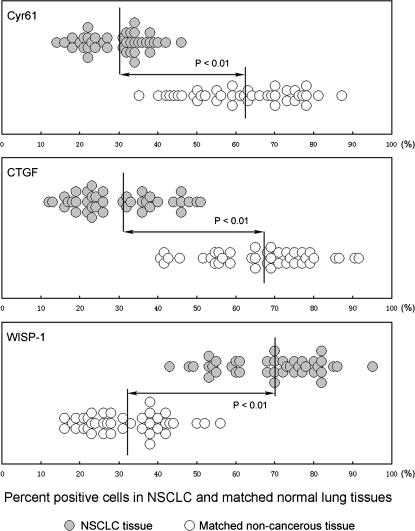
Expression patterns of Cyr61, CTGF, and WISP-1 protein in NSCLC and matched normal lung tissues. The graph depict distributions of specimens according to the percentage of cells positive for Cyr61, CTGF, and WISP-1 in 60 pairs of NSCLC and matched non-cancerous lung tissues. P values (paired-samples t-test) are listed suggesting the difference between NSCLC samples versus matched normal tissues.

**Figure 3:**
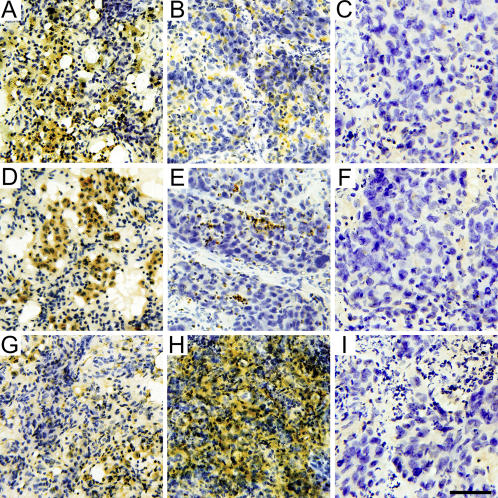
Representative immunohistochemistry result, staining for Cyr61, CTGF, and WISP-1 in NSCLC and matched normal lung tissues. In non-cancerous lung tissues from patients with NSCLC, intense Cyr61 immunoreactivity (brown) is observed in the cytoplasm of cells as well as extracellular space (A). In corresponding NSCLC tissues, Cyr61 immunoreactivity is less (B) compared with normal lung tissue. Similar to Cyr61, many CTGF-positive cells (brown) are visible in non-tumor lung tissues (D), and positive signals are only sparsely distributed in NSCLC tissues (E). WISP-1 immunohistochemistry, however, displays a different expression pattern. WISP-1 immunoreactivity (brown) is barely detected in normal lung tissues from NSCLC patients (G) but is markedly increased in NSCLC tissues (H). C, F, and I are negative controls of Cyr61, CTGF, and WISP-1, respectively. All slides are counterstained with hematoxylin (blue). Scale bar = 60 µm.

Immunohistochemistry was used to monitor the protein expression of Cyr61, CTGF, and WISP-1 in the 60 pairs of lung tissues. Paired-samples t-test indicated that protein expression of Cyr61 (P<0.001) and CTGF (P<0.001) was dramatically downregulated in cancer tissues compared with their normal counterparts (Figure 2). Additionally, significant upregulation of WISP-1 was found in the cancer samples (P<0.001) in comparison with matched normal tissues (Figure 2). Representative photographs showed that Cyr61 was highly expressed in the cytoplasm of lung epithelial cells in the normal lung tissues of patients with lung carcinoma (Fig. 3A), but levels were remarkably decreased in the cancerous counterparts (Fig. 3B). Notably, the downregulation of Cyr61 is mainly due to the large reduction of Cyr61 expression level in individual cells rather than a decreased number of Cyr61-positive lung epithelial cells. CTGF displayed higher intensity of staining in the cytoplasm of normal lung epithelial cells (Fig. 3D) than that in lung cancer cells (Fig. 3E); the ratio of CTGF-positive to -negative cells in cancer tissues was clearly lower in comparison with the corresponding normal lung tissues. Although expression of WISP-1 was low in the cytoplasm of epithelial cells derived from normal lung tissues (Fig. 3G), it was remarkably increased in matched lung-cancer tissues (Fig. 3H). Taken together, the result of immunohistochemistry paralleled those of real-time RT-PCR.

### Relationship between expression of Cyr61 in lung cancers and the clinical and pathological features of the individuals

Univariate analysis proved that a significant association existed between expression of *Cyr61 versus* tumor stage, metastasis, histological subtype, smoking and family history (Table 3). Level of *Cyr61* expression was decreased significantly in high stage tumors compared to low stage tumors (P = 0.014). Multiple comparison (LSD t-test) analysis showed that expression of *Cyr61* between tumors of stages I and III were significantly different (P = 0.004); but no significant difference in *Cyr61* expression occurred in NSCLC samples comparing either stages I and II (P = 0.063) or stages II and III (P = 0.17). Histological analysis showed that a significant difference existed among SC, AC, adeno-squamous cell carcinoma (ASC) and other pathological type in level of expression of *Cyr61* (P = 0.001). Moreover, multiple comparison (LSD t-test) demonstrated that expression of *Cyr61* between SC *versus* ASC (P = 0.009) or SC *versus* other pathological type (P = 0.001) was significantly different. Similarly, significant differences of *Cyr61* expression were also observed between AC *versus* ASC (P = 0.015) or AC *versus* other pathological type (P = 0.002). Smoking was associated with low expression of *Cyr61* (P = 0.009). In contrast, expression of *Cyr61* was not correlated with tuberculosis, gender, age, and tumor size (Table 3).

**Table 3:** Relationship between levels of *Cyr61*, *CTGF*, and *WISP-1* mRNA in lung cancer and the clinical and pathological features of these individuals.

Factors	N	*Cyr61*		*CTGF*		*WISP-1*	
		Mean±SD*	P**	Mean±SD	P value	Mean±SD	P
Family history†			0.027		0.038*		0.767
No	48	−1.72±3.30		2.69±2.37		2.68±2.99	
Yes	12	−4.07±2.79		0.59±3.20		2.96±2.68	
Metastasis			0.003		0.039*		0.408
No	29	−0.91±3.05		1.81±3.00		3.06±2.98	
Yes	31	−3.39±3.15		0.15±3.12		2.43±2.87	
Smoke			0.009		0.017*		0.610
No	23	−0.78±3.25		1.76±3.08		2.49±2.11	
Yes	37	−3.06±3.09		−0.21±2.93		2.89±3.33	
Tumor stage			0.014		0.040*		0.969
I	17	−0.65±3.08		2.48±2.55		2.66±2.01	
II	25	−2.03±3.28		0.69±2.68		2.84±3.45	
III	18	−3.86±2.96	0.014	−0.09±3.87	0.040*	2.65±2.97	0.969
Histology			0.001		0.005**		0.017
SC‡	25	−3.32±2.42		2.49±2.16		1.85±2.74	
AC^§^	15	−3.35±3.65		0.78±2.57		2.39±2.52	
ASC^¶^	10	−0.31±3.36		−0.17±4.57		5.19±3.47	
Other^$^	10	0.49±2.63		−1.19±2.88		3.01±2.12	
Tuberculosis			0.719		0.324		0.299
No	51	−2.13±3.50		1.18±3.14		2.57±2.94	
Yes	9	−2.56±2.16		0.05±3.22		3.67±2.74	
Gender			0.729		0.849		0.358
Male	45	−2.28±3.55		0.96±3.41		2.93±3.19	
Female	15	−1.93±2.59		1.14±2.29		2.13±1.79	
Tumor size			0.988		0.994		0.141
≤100 cm^3^	26	−2.09±3.79		1.08±3.53		2.53±2.78	
100 cm^3^–	10	−2.51±4.16		0.87±2.88		4.02±3.63	
200 cm^3^–	8	−2.03±1.83		0.78±2.55		0.94±1.82	
≥300 cm^3^	16	−2.24±2.72		1.10±3.19		3.16±2.82	
Age			0.089		0.801		0.047
35–	11	−1.29±4.01		1.16±3.47		4.68±3.85	
50–	34	−3.01±3.00		1.13±2.95		2.30±2.63	
≥65	15	−1.00±3.15		0.43±3.53		2.29±2.29	

† All patients included in this study were asked whether their living or dead first-degree relatives (parents, siblings, children) had been affected by a lung malignancy.

‡ squamous-cell carcinoma; ^§^adenocarcinoma; ^¶^adenosquamous-cell carcinoma; ^$^including other pathological types except for squamous-cell carcinoma, adenocarcinoma, adeno-squamous cell carcinoma.

* The value is the relative levels of normalized target genes by β-*actin* in cancer versus matched normal samples.

** P<0.05 and P<0.01 are set for significant and highly significant difference, respectively.

### Relationship between expression of *CTGF* in lung cancers and the clinical and pathological features of the individuals

Statistical analysis revealed that expression of *CTGF* was strongly associated with some clinical features of NSCLC, including tumor stage, metastasis, histology, smoking, and family history (Table 3). Level of *CTGF* was decreased significantly in high-stage tumors compared to low-stage tumors (P = 0.040). Moreover, significant difference of *CTGF* expression existed between tumors, stage I *versus* stage III (P = 0.013) and stage II *versus* stage III (P = 0.024); however, no significant statistical difference was observed between tumors, stage I and II (P = 0.077). For metastasis, the expression of *CTGF* in metastatic NSCLC was significantly lower than in non-metastatic NSCLC (P = 0.039); significantly, smoking was associated with a prominent suppression of *CTGF* expression in NSCLC (P = 0.017) (Table 3). Additionally, a significant difference occurred among SC, AC, ASC and other pathological types in the level of CTGF (P = 0.005). In addition, level of *CTGF* in the patients was significantly lower than those whose family members did not have cancer (P = 0.038). However, gender, tumor size, age and histology of tuberculosis showed no significant correlations with the expression of *CTGF* (Table 3).

### Relationship between expression of *WISP-1* in NSCLC and the clinical and pathological features of the individuals

Statistical analysis showed that expression of *WISP-1* was significantly associated with tumor histology, as well as age of NSCLC patients at diagnosis (Table 3). One-way ANOVA analysis demonstrated that significant differences existed among SC, AC, ASC and other histological type in their expression of *WISP-1* (P = 0.017). Level of *WISP-1* in ASC was much higher than those in either SC (P = 0.002) or AC (P = 0.015). Moreover, younger patients (ages, 35–50) had a significantly higher level of *WISP-1* in their tumors than older individuals (ages, 50–65 or ≥65) (P = 0.018 and P = 0.036, respectively). In contrast, other clinical parameters (family history, metastasis, history of smoking, tuberculosis, gender, tumor type, and tumor size) were not associated with expression of *WISP-1*.

### Expression of *Cyr61*, *CTGF*, *WISP-1* genes and clinical outcome of NSCLC

**Figure 4:**
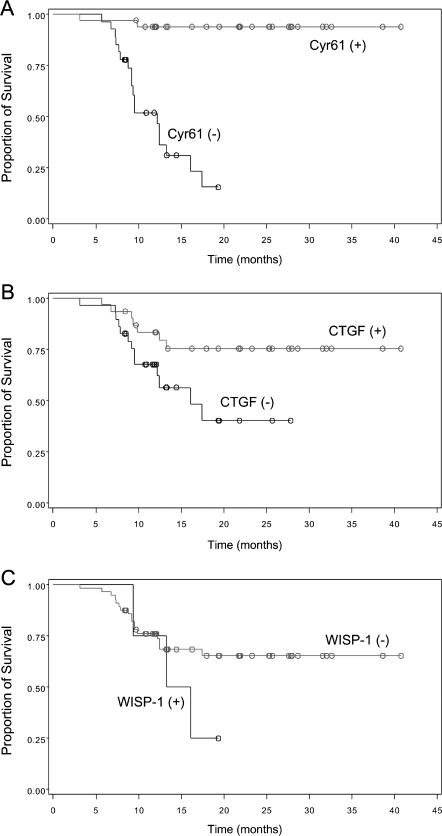
Survival curves from the time of diagnosis for NSCLC patients with either high (+) or low (−) expression of *Cyr61* (A), *CTGF* (B), and *WISP-1* (C).

Univariate survival analysis showed that the expression of *Cyr61*, *CTGF*, metastasis, and smoking were significantly associated with survival (Table 4). Kaplan-Meier curves suggested that patients with the high-expression of *Cyr61* showed a significantly extended mean survival time in comparison with the other patients (Fig. 4A) (P = 0.001). Similarly, high levels of *CTGF* were associated with prolonged survival (Fig. 4B) (P = 0.031). In contrast, no significant association of *WISP-1* expression and survival was noted (Fig. 4C) (P = 0.214). After controlling the clinical and pathological characteristics (age, gender, metastasis, smoke, tumor stage, tuberculosis, tumor size), multivariate (Cox regression) survival analysis was conducted to evaluate the potential effects of the three CCN genes on cancer prognosis. The result showed that both *Cyr61* and *CTGF* were significantly independent positive prognostic factors for survival of patients with NSCLC, and the relative risk was 0.047 for *Cyr61* and 0.0357 for *CTGF* (Table 5). However, no significant association occurred between *WISP-1* expression and survival (Table 5).

**Table 4:** Univariate survival analysis of expression of *Cyr61, CTGF* and *WISP-1* mRNA in lung cancer and the clinical and pathological of these individuals.

Factors	N	Events	Mean Survival (Months)	SE	P‡
**Gender**					**0.4012**
Male	45	15	8.5280	0.5314	
Female	15	5	15.1274	0.5635	
**Age(year)**					**0.0598**
≤59	31	7	15.8039	0.7233	
>59	29	13	11.4247	0.4792	
**Metastasis**					**0.0001**
No	29	2	15.8548	0.3963	
Yes	31	18	12.2135	0.8556	
**Family history** †					**0.6451**
No	48	17	14.9562	0.5524	
Yes	12	3	6.6275	0.6240	
**Smoke**					**0.0387**
No	23	10	12.6333	0.9882	
Yes	37	10	15.5517	0.5926	
**Tumor stage**					**0.4774**
I	17	0	Not reached	Not reached	
II	25	13	13.7476	0.6353	
III	18	5	9.1541	0.1925	
**Tuberculosis**					**0.7314**
No	51	17	14.7840	0.6068	
Yes	9	3	11.2774	0.8807	
**Tumor size**					**0.3670**
≤100 cm^3^	26	0	Not reached	Not reached	
100 cm^3^	10	13	13.7517	0.6107	
200 cm^3^	8	3	15.5655	1.4845	
≥300 cm^3^	16	4	8.9278	0.8053	
***Cyr61***					**0.0001**
Low expression	27	18	9.6648	0.2857	
High expression	33	2	11.9306	0.8388	
***CTGF***					**0.0313**
Low expression	29	13	12.4020	0.3870	
High expression	31	7	13.6814	0.9023	
***WISP-1***					**0.4008**
Low expression	30	12	13.6041	0.6590	
High expression	30	8	15.0882	0.8590	

† All patients included in this study were asked whether their living or dead first-degree relatives (parents, siblings, children) had been affected by a lung malignancy.

‡ P<0.05 and P<0.01 are set for significant and highly significant difference, respectively.

**Table 5:** Cox regression analysis of *Cyr61*, *CTGF*, and *WISP-1* mRNA in lung cancer.

Gene	N	RR	95%CI†	P‡
*Cyr61*	60	0.047	0.010–0.216	0.0001
*CTGF*	60	0.357	0.137–0.933	0.0356
*WISP-1*	60	1.597	0.537–4.748	0.4000

† CI: confidence interval

‡ P<0.05 and P<0.01 are set for significant and highly significant difference, respectively.

## Discussion

Cyr61, CTGF, and WISP-1 play important roles in cell proliferation, migration and differentiation. Their expression appears to be regulated differently in various types of tumors. To explore the correlation between expression of CCN genes and NSCLC, we used real-time PCR and immunohistochemistry to evaluate the mRNA and protein levels of these three genes in NSCLC and their matched normal lung tissues. Downregulation of *Cyr61* and *CTGF* and upregulation of WISP-1 occurred in the NSCLC samples compared to their normal counterparts, suggesting that these molecules might be associated with tumor formation and progression in NSCLC.

Cyr61 is the first cloned member of the CCN family and its regulatory roles in tumor cells have been widely reported in many types of cancers. In breast cancers, Cyr61 is overexpressed and can stimulate tumor progression [24]–[27]. A gastric adenocarcinoma cell line became more tumorigenic when the cells were genetically engineered to express high levels of Cyr61 [16]. Expression of Cyr61 was high in rhabdomyosarcomas and cell lines derived from malignant melanomas, colon adenocarcinomas, and bladder papillomas [16], [28]. Malignant gliomas often have high levels of Cyr61 associated enhanced tumorigenicity mediated through the integrin-linked kinase signaling pathway [29]. Upregulation of Cyr61 expression was recently identified in peritoneal metastases from human pancreatic cancer [30]. Paradoxically, Cyr61 appears to have the opposite role in lung cancer. We have reported that Cyr61 was downregulated in four of 5 samples of lung cancer, and lung cancer cells stably transfected with a Cyr61 expression vector were less tumorigenic than the vector alone transfected control cells [19]. Our further studies showed that forced expression of Cyr61 in lung cancer cells resulted in their cell-cycle arrest in G1 phase mediated by p53 [6]. Here, we found that expression of *Cyr61* is decreased in NSCLC samples compared to their matched controls, which strongly supports our former hypothesis. Cyr61 was also reported to inhibit growth of prostate cancer [31], endometrial cancer [32] and leiomyomas [33]. Taken together, the data suggested that Cyr61 might behave as a tumor suppressor under certain circumstances in several tissue types, including NSCLC.

CTGF was identified as a mitogen found in the conditioned medium of human umbilical vein endothelial cells [34]. It encodes a protein of 349 amino acids with 43% sequence identity to Cyr61, and all 38 cysteines in CTGF and Cyr61 are completely conserved. CTGF was found to be overexpressed in mammary tumors [26], [35], melanomas [36], pancreatic cancers [37], sarcomas including chondrosarcomas [38], [39]; while an inverse correlation has been reported between the malignant phenotype and the level of CTGF expression in fibroblasts and endothelial cell tumors [40]. In our experiments, we found that the level of expression of *CTGF* in NSCLCs was lower than in the matched normal lung samples, implying its potential tumor-suppressing function. Consistent with our finding, a recent study by Chien *et al.* [20] showed that CTGF suppressed lung cancer cell growth by induction of p53, as well as, by inhibition of insulin-like growth factor-I dependent Akt phosphorylation and epidermal growth factor-dependent extracellular signal-regulated kinase 1/2 phosphorylation.

On the other hand, we found that *WISP-1* was overexpressed in NSCLC samples compared to their normal lung tissue counterparts, suggesting that WISP-1 might act as an oncoprotein in NSCLC. WISP-1 was identified as a gene that was upregulated in Wnt-1 transformed C57 MG mouse mammary epithelial cells; it has complete conservation of all 38 cysteine residues with those of Cyr61 and CTGF [10]. WISP-1 has been associated with either enhancing or inhibiting growth of tumors. For example, it is strongly expressed in human breast and colon cancers [10]. Forced overexpression of WISP-1 in normal rat kidney fibroblasts (NRK-49F) was sufficient to induce their transformation [11]. In contrast, WISP-1 expression was inversely correlated with proliferation, metastasis and growth of melanoma cells [41], [42]. Additionally, our result that upregulation of WISP-1 was positively correlated with lung cancer metastasis was consistent with findings from a mouse model [21]. Paradoxically, Soon *et al.* found that *in vitro* overexpression of WISP-1 decreased motility of lung cancer cells [22]. Since WISP-1 is a secreted protein and functions mainly by interaction with ECM, the difference in findings might reflect the difference in ECM *in vivo* and *in vitro*.

Univariate statistical analysis further disclosed that low levels of either *Cyr61* or *CTGF* were related with the progression of NSCLC, and the downregulation of *Cyr61* and *CTGF* was more notable in patients with family history than those without family history. These data implied that Cyr61 and CTGF might be tumor suppressor genes in lung cancer. Through multiple linear regression analyses, moreover, we found that clinical features are closely associated with levels of expression of *Cyr61*, *CTGF* and *WISP-1*. Expression level of *Cyr61* in 71% lung cancer samples was determined by eight independent variables, mainly by AC, SC and age. Levels of *CTGF* in NSCLCs were also markedly associated with clinical features, but with some differences. Gender seemed to play a role in expression of *CTGF*, but not *Cyr61*. Sex hormone might regulate expression of *CTGF*, but more work is needed to confirm this impression. Statistical analysis also revealed that levels of these molecules were correlated with one another, suggesting that their regulation may relate to similar signaling pathways. Furthermore, levels of *Cyr61*, *CTGF*, and WISP-1 were tightly related to pivotal clinical and prognostic features of NSCLC. Perhaps, examining the levels of these three molecules either in the primary tumor or in the malignant cells from sputa of patients may help guide therapy.

In summary, our study examined the three CCN molecules (*Cyr61*, *CTGF*, and *WISP-1*) and found correlations between their levels and clinical features of NSCLCs. Although the detailed mechanism remains to be investigated, our results might provide new parameters for diagnosis and prognosis of NSCLC.
